# Importance of nondiffusive transport for soil CO_2_ efflux in a temperate mountain grassland

**DOI:** 10.1002/2014JG002788

**Published:** 2015-03-24

**Authors:** Marilyn Roland, Sara Vicca, Michael Bahn, Thomas Ladreiter‐Knauss, Michael Schmitt, Ivan A. Janssens

**Affiliations:** ^1^ Department of BiologyUniversity of AntwerpWilrijkBelgium; ^2^ Institute of EcologyUniversity of InnsbruckInnsbruckAustria

**Keywords:** soil CO_2_ transport, diffusion, flux‐gradient approach, chamber measurements, solid‐state sensors, pressure pumping

## Abstract

Soil respiration and its biotic and abiotic drivers have been an important research topic in recent years. While the bulk of these efforts has focused on the emission of CO_2_ from soils, the production and subsequent transport of CO_2_ from soil to atmosphere received far less attention. However, to understand processes underlying emissions of CO_2_ from terrestrial ecosystems, both processes need to be fully evaluated. In this study, we tested to what extent the transport of CO_2_ in a grassland site in the Austrian Alps could be modeled based on the common assumption that diffusion is the main transport mechanism for trace gases in soils. Therefore, we compared the CO_2_ efflux calculated from the soil CO_2_ concentration gradient with the CO_2_ efflux from chamber measurements. We used four commonly used diffusion‐driven models for the flux‐gradient approach. Models generally underestimated the soil chamber effluxes and their amplitudes, indicating that processes other than diffusion were responsible for the transport of CO_2_. We further observed that transport rates correlated well with irradiation and, below a soil moisture content of 33%, with wind speed. This suggests that mechanisms such as bulk soil air transport, due to pressure pumping or thermal expansion of soil air due to local surface heating, considerably influence soil CO_2_ transport at this site. Our results suggest that nondiffusive transport may be an important mechanism influencing diel and day‐to‐day dynamics of soil CO_2_ emissions, leading to a significant mismatch (10–87% depending on the model used) between the two approaches at short time scales.

## Key Points

- We compared chamber soil CO_2_ efflux and soil CO_2_ concentrations
- We found correlations between transport rates and wind speed and irradiation
- Nondiffusive gas transport should be considered at any site

## 1 Introduction

Soil CO_2_ efflux is the largest source of CO_2_ from terrestrial ecosystems; annually, approximately 98 Pg CO_2_ is emitted from soils [*Bond‐Lamberty and Thomson*, 2010]. Although in recent years the number of studies on soil CO_2_ fluxes has largely increased, and high‐frequency measurements have provided new insights in short‐term dynamics of CO_2_ efflux [see, e.g., *Vargas et al*., 2011], the efflux of CO_2_ from soil to atmosphere and its biotic and abiotic drivers remain an important topic of debate [e.g., *Subke and Bahn*, 2010]. A major reason for this ambiguity is related to the fact that in the short term, soil CO_2_ efflux does not equal to soil CO_2_ production (also termed soil respiration, the sum of microbial and root respiration). Our limited knowledge of the CO_2_ transport through the soil restricts our understanding of the role of the various abiotic and biotic controls on soil respiration [*Phillips et al*., 2011; *Subke and Bahn*, 2010].

Most often, soil CO_2_ efflux is measured using soil chambers, but since the early 2000s, solid‐state CO_2_ sensors that measure soil CO_2_ concentrations at different soil depths are becoming more common. In contrast to soil chambers, solid‐state CO_2_ sensors allow continuous high‐frequency measurements of the CO_2_ gradient with minimal disturbance of the natural conditions, such as air pressure or wind velocity [*Pingintha et al*., 2010; *Tang et al*., 2003] and soil microclimate. For this reason, estimation of the soil CO_2_ efflux from soil CO_2_ concentrations, the so‐called flux‐gradient approach, is rapidly gaining popularity [e.g., *Hirano et al*., 2003; *Jassal et al*., 2005; *Jassal et al*., 2004; *Pumpanen et al*., 2008; *Tang et al*., 2003; *Tang et al*., 2005; *Turcu et al*., 2005; *Vargas and Allen*, 2008a; *Vargas and Allen*, 2008b]. This method uses Fick's law of diffusion (equation (1)) to compute soil CO_2_ efflux and thus implies the assumption that diffusion is the only transport mechanism for CO_2_ through the soil. Potential effects of, for example, air pressure differences (following advection or wind shear) are often neglected. Soil CO_2_ efflux *F* is calculated via the flux‐gradient method as

(1) $$F=-D_{s}\frac{\partial C}{\partial z}$$

where *D_s_* is the effective diffusion coefficient (see Materials and Methods for more detailed information) and *C* is the CO_2_ concentration at depth *z* in the soil.

Gas diffusion in soils differs from that in free air, because solid and liquid obstacles reduce the cross‐sectional area and increase the mean path length for the diffusing molecules [*Sallam et al*., 1984; *Werner et al*., 2004]. Soil properties such as water content, texture, and bulk density therefore determine the rate of diffusion [*Moldrup et al*., 1999; *Pumpanen et al*., 2003; *Vargas et al*., 2010]. Reliable estimates for the diffusion coefficient are of critical importance when using Fick's law to estimate soil CO_2_ efflux from soil CO_2_ concentrations. Several commonly used models have been proposed to calculate the diffusion coefficient, all of them depending primarily on the air‐filled pore space and thus varying inversely with soil water content [*Jassal et al*., 2005].

Generally, it is assumed that provided that a good estimate of the diffusion coefficient is available, the soil CO_2_ concentration gradient directly translates to soil CO_2_ efflux. However, there is limited evidence that nondiffusive transport, such as pressure pumping (summarized in *Takle et al*. [2004]) and advective transport due to heating of the soil surface [*Ganot et al*., 2014], may strongly influence soil CO_2_ emissions at the time scale of seconds. Air pressure at the soil surface fluctuates whenever turbulent air moves over the surface and enhances the exchange of gases at shallow depths [*Kimball and Lemon*, 1971]. This bulk air gas transport is increased with increasing permeability of the soil, corresponding to a decreasing soil water content, and by thermal advection. As the soil water content decreases, the air‐filled porosity increases, enhancing both diffusive and nondiffusive transports.

While the potential relevance of nondiffusive transport has been mostly addressed at a very short time scale, it is important to test if it plays a role at half‐hourly time steps, which is the highest time resolution typically achieved in soil respiration studies. So far, few such studies have tested how well rates of chamber‐measured soil CO_2_ efflux and those estimated from the flux gradient approach compare across the season [*Riveros‐Iregui et al*., 2008], when nondiffusive transport may intermittently decouple fluxes derived from these two approaches.

We aim to deduce the importance of nondiffusive transport for soil CO_2_ effluxes by comparing two in situ measurement methods (chamber and soil concentration gradients). Given that the CO_2_ efflux is derived from the concentration gradient using diffusion models, the discrepancy between CO_2_ effluxes from both measurements can be an indication for nondiffusive gas transport.

We tested the hypotheses that the role of nondiffusive transport (i.e., the mismatch between the two approaches) increases with increasing radiation and wind speed and with decreasing soil moisture.

## 2 Materials and Methods

### 2.1 Site Description

The study was carried out in a mountain meadow at Kaserstattalm, Neustift, in the Austrian Central Alps [cf. *Bahn et al*., 2009]. Mean annual temperature and precipitation are 3.0°C and 1097 mm, respectively. The meadow is located at 1820 m above sea level and is fertilized with manure in spring, cut once in late July or early August, and lightly grazed in September. The dominating plant species include the grasses *Anthoxanthum odoratum* L. and *Festuca rubra* L. and the forbs *Alchemilla vulgaris* L., *Leontodon hispidus* L., and *Trifolium repens* L. The soil is a cambisol on siliceous bedrock with a topsoil pH of 5.5. The soil texture is 43% sand, 47% silt, and 11% clay; the bulk density is 860 kg m^−3^. The meadow is characterized by a comparatively high productivity and high soil respiration rates, typical for non–water‐limited central European mountain meadows [*Bahn et al*., 2010; *Schmitt et al*., 2010].

### 2.2 Measurements

Soil CO_2_ concentration measurements were made during the growing season of 2009 using Vaisala CARBOCAP solid‐state CO_2_ sensors (model GMT 221, Vaisala, Helsinki, Finland) at depths of 10 and 5 cm and the Li‐8150 system (Li‐Cor, Lincoln, NE, USA) at 0 cm. At the same depths, soil temperature (averaging soil thermocouple probe TCAV; Campbell Scientific) and moisture (ML2x; Delta‐T Devices, Cambridge, UK) were measured, and incident photosynthetically active radiation (PAR) (BF2H; Delta‐T Devices) was measured above the canopy at 2 m height. Continuous recordings at 0.05 Hz were averaged and half‐hourly values recorded using an automated station (CR10X; Campbell Scientific). Values of soil CO_2_ concentration were corrected for temperature and pressure using the ideal gas law according to the manufacturer (Vaisala, Helsinki, Finland). Soil CO_2_ efflux at the soil surface was measured using an automated soil respiration system (Li‐8100 and Li‐8150; Li‐Cor, Lincoln, NE, USA) over measurement intervals of 2 min. The chambers were white to minimize heating. Possible pressure changes due to a Venturi effect are largely eliminated by the design of the vent used with the chamber [*Xu et al*., 2006].

### 2.3 CO_2_ Diffusion Through the Soil

#### 2.3.1 Effective Diffusivity

Using Fick's law (equation (1)) to calculate soil CO_2_ efflux from the soil CO_2_ concentration gradient requires good estimates of the diffusion coefficient *D_s_* (m^2^ s^−1^), also named effective diffusivity. *D_s_* can be estimated as

(2) $$D_{s}=D_{a}\xi$$

where *D_a_* (m^2^ s^−1^) is the CO_2_ diffusion coefficient in free air and *ξ* (m^3^ m^−3^) is the so‐called tortuosity factor, the product of the air‐filled porosity *ε*_*a*_ and the tortuosity *τ* [*Jassal et al*., 2005; *Jury et al*., 1991]. It accounts for the increase in path length and decrease in cross‐sectional area in soils.

The variation of *D_a_* with temperature and pressure is given by

(3) $$D_{a}=D_{a0}{\left(\frac{T}{T_{0}}\right)}^{1.75}\left(\frac{P}{P_{0}}\right)$$

with *T* as the temperature (K), *P* as the air pressure (Pa), and *D*_*a*0_ as a reference value at *T*_0_ (293.15 K) and *P*_0_ (1.013 × 10^5^ Pa), given as 1.47 × 10^−5^ m^2^s^−1^ [*Jones*, 1992].

The air‐filled pore space *ε_a_* (m^3^ m^−3^) is defined as the difference between total porosity *ϕ* and the volumetric water content *θ* (m^3^ m^−3^) of the soil.

(4) $$\varepsilon_{a}=\;\varphi -\theta$$

Several relationships between *ξ* and *ε_a_* have been proposed to d or *ξ*. Here we compare four of the most commonly used models:

(5) $$\xi =0.66\varepsilon_{a}$$

[*Penman*, 1940],

(6) $$\xi ={\varepsilon_{a}}^{1.5}$$

[*Marshall*, 1959],

(7) $$\xi =\;\frac{{\varepsilon_{a}}^{10/3}}{\varphi^{2}}$$

[*Millington and Quirk*, 1961],

(8) $$\xi =\;\varphi^{2}{\left(\frac{\varepsilon_{a}}{\varphi }\right)}^{\beta S}$$

[*Moldrup et al*., 1999].

In equation (8), *S* is the percentage of mineral soil with particle size >2 µm (*S* = 0.8 for our study site), and *β* is a constant equal to 2.9. The total porosity at the site is 0.57 m^3^ m^−3^. Note that in all four models, the air‐filled pore space and thus total porosity, is a key factor determining the calculated efflux. Measurements of porosity on soil cores are often subject to uncertainties, especially on heterogeneous soils, and 10% uncertainty in estimated porosity translates into 27–80% uncertainty in calculated efflux (depending on the model used). For this uncertainty, corresponding to a porosity range of 0.51 to 0.63 m^3^ m^−3^ at our study site, the *Millington and Quirk*'s[1961] model is the most sensitive (up to 80% uncertainty on the efflux), while the *Penman*'s [1940] model is the least sensitive (27% uncertainty on the efflux).

#### 2.3.2 Apparent Diffusivity, *D*_app_

The combination of chamber measurements and solid‐state sensors allows the calculation of the diffusion coefficient solely from measurements. To make a distinction with the effective diffusivities *D_s_* calculated from equations (5), (6), (7), (8), we refer to the empirically obtained diffusivity coefficient as “apparent diffusivity, *D*_app_.” To calculate this, we used the difference between the CO_2_ concentration measurements at 5 and 0 cm depth, which should capture the most productive zone of the grassland rhizosphere. When calculating *D*_app_ from Fick's law (equation (1)), it is necessary to account for the temperature gradient in the soil that causes changes in the soil air density and thus in the absolute gas concentration [*Kowalski and Argueso*, 2011]. When this concentration gradient is proportional to the gradient in air density, it will not result in diffusional transport and yield wrong fluxes. Therefore, we expressed CO_2_ concentration as a molar fraction and accounted for the air density.

(9) $$F=-\rho D_{s}\frac{\partial C}{\partial z}$$

where *F* is the mass flux (kg m^−2^ s^−1^), *ρ* is the density of air (kg m^−3^), and *C* is the relative CO_2_ concentration (dimensionless) at depth *z* (m) in the soil. *D*_app_ can then be calculated by rearranging this equation:

(10) $$D_{\text{app}}=-F_{ch}\frac{\partial z}{\rho \partial C}$$

It is important to note that if the chamber soil CO_2_ efflux results from transport mechanisms other than diffusion, these are also comprised by *D*_app_.

## 3 Results

### 3.1 Measurements of Soil CO_2_ Concentrations and Soil CO_2_ Efflux

**Figure 1:**
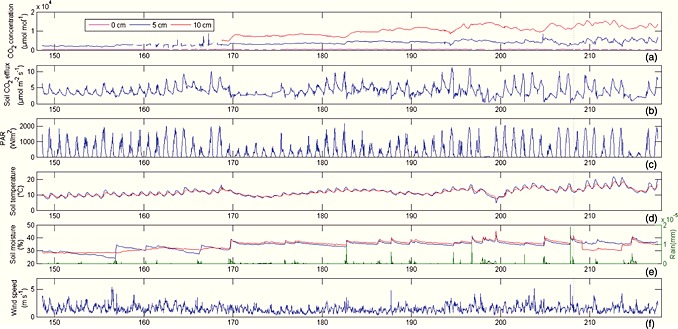
Time series from 28 May to 4 August 2009 of half hourly measurements of (a) soil CO_2_ concentrations at 0, 5, and 10 cm depths; (b) soil CO_2_ efflux from automated chambers; (c) photosynthetically active radiation (PAR); (d) soil temperature at 5 and 10 cm depth; (e) soil water content and rainfall; and (f) wind speed on a mountain meadow at Kaserstattalm, Neustift, Austria.

Chamber‐based measurements of soil CO_2_ efflux showed most variability at daily time scales, with an amplitude apparently in phase with variations in temperature, light (PAR), and wind speed (Figure 1). Soil CO_2_ concentrations measured with solid‐state sensors at 5 cm and 10 cm depths and measured with the Li‐8150 system at 0 cm depth were much less variable both at seasonal and daily time scales. As the CO_2_ gradient is a driver of the CO_2_ efflux, we expected a clear relationship between the two types of measurements, but we found that the soil CO_2_ concentration gradients from 5 to 0 cm and from 10 to 5 cm depths were uncoupled from the efflux measured with the chambers. Slopes of the correlations between the concentration measurements at 10 cm and at 5 cm depth with the fluxes were not significantly different from zero (*p* = 0.92 and *p* = 0.33, respectively).

### 3.2 Calculation of Soil CO_2_ Efflux With Flux‐Gradient Approach

**Figure 2:**
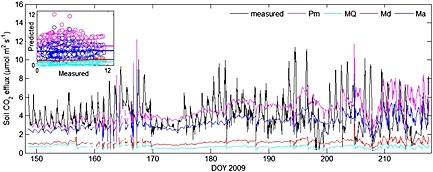
Comparison of measured and modeled soil CO_2_ effluxes. The black line shows the soil CO_2_ efflux measured by the soil chambers and the colored lines are the effluxes calculated using four different models: Pm: *Penman* [1940], MQ: *Millington and Quirk* [1961], Md: *Moldrup et al*. [1999], and Ma: *Marshall* [1959].

Using Fick's law (equation (1)), we calculated the soil CO_2_ efflux from the measured soil CO_2_ concentrations at 5 and 0 cm depths and the modeled diffusivity. We will refer to this calculated flux as the solid‐state CO_2_ efflux as distinct from the chamber CO_2_ efflux. Note that we did not use the concentrations at 10 cm depth in this analysis, as the peak rhizosphere activity is assumed to be in the upper 5 cm of the soil. The four different models (see equations (5), (6), (7), (8)) to estimate the effective diffusivity, *D_s_*, revealed large differences in estimated soil CO_2_ efflux. Using the *Penman* [1940] and *Marshall*'s [1959] models for *D_s_* yielded flux estimates within the range of the chamber measured fluxes, while the *Moldrup et al*. [1999] and *Millington and Quirk*'s [1961] models for *D_s_* were both considerably lower than the chamber CO_2_ effluxes (Figure 2). Importantly, none of the four models was able to reproduce the large daily variation in soil CO_2_ efflux that was observed with the chamber‐based measurements (Figure 2).

The diurnal course of the solid‐state fluxes clearly followed that of the CO_2_ concentrations, soil temperature, and soil moisture content, while the course of the chamber fluxes followed that of wind speed and PAR. As a result, the daily peaks of solid‐state fluxes lagged the chamber fluxes with approximately 4 h.

### 3.3 Effective and Apparent Diffusivities, *D_s_* and *D*_app_

**Figure 3:**
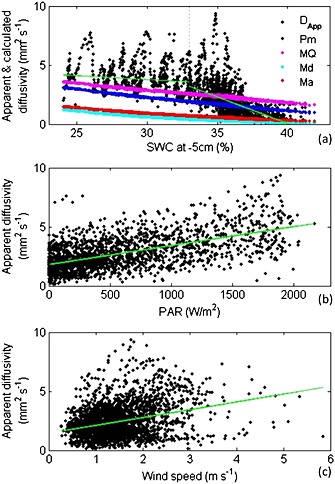
(a) Apparent diffusivity (*D*_app_, black dots) and modeled diffusivity (*D_s_*, colored dots) as a function of the soil water content (SWC) at 5 cm depth. The green lines are the regression lines of *D*_app_ versus SWC < 33% (*y* = −0.06 × +5.66, *R* ^2^ = 0.02, *p* < 0.01) and *D*_app_ versus SWC > 33% (*y* = −0.45 × +18.06, *R* ^2^ = 0.30, *p* < 0.01). (b) *D*_app_ as a function of photosynthetically active radiation (PAR). The green line is the regression line (*y* = 0.002 × +1.81, *R* ^2^=0.35, *p* < 0.01). (c) *D*_app_ as a function of wind speed. The green line is the regression line (*y* = 0.66 × +1.46, *R* ^2^=0.10, *p* < 0.01).

By inverting Fick's law and combining the chamber‐based CO_2_ efflux measurements and the solid‐state CO_2_ concentration measurements, we calculated *D*_app_ and compared this with the four commonly used models to calculate *D_s_*. *D_s_* calculated from all four models strongly differed from *D*_app_ (Figure 3a). While *D_s_* linearly decreases with increasing soil moisture, *D*_app_ revealed a different pattern (Figure 3a). Only for soil water contents above 33%, *D*_app_ markedly decreased with increasing soil moisture. At soil water contents below 33%, *D*_app_ was unrelated to soil moisture and substantially varied even at similar values of soil water content.

**Figure 4:**
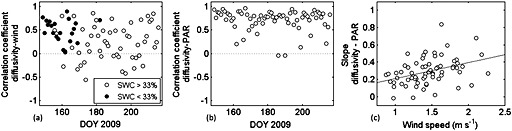
(a) Correlation coefficients of 24 h fits (using 48 half‐hourly values for each fit) of apparent diffusivity (*D*_app_) and wind speed. The open circles are the days with soil water content below 33%; the full circles are the days with soil water content above 33%. (b) Correlation coefficients of 24 h fits of *D*_app_ and of photosynthetically active radiation (PAR). (c) Slopes of 24 h fits of *D*_app_ and PAR plotted against wind speed. The black line is the regression line (*y* = 0.19 × +0.02, *R* ^2^=0.15, *p* < 0.01).

Because soil moisture is the primary determinant of gas diffusion in soils (see equations (4), (5), (6), (7), (8)), the high variation of *D*_app_ at similar soil moisture, especially at soil moisture levels below 33%, suggests that processes other than diffusion influence the transport of CO_2_ from soil to atmosphere. Therefore, we tested whether light (PAR), soil temperature, and wind speed affected *D*_app_. Figures 3b and 3c show that *D*_app_ indeed responded positively to variations in light and wind speed. We found no correlation between the soil temperature and *D*_app_, and the temperature dependence of *D_s_* was very weak (data not shown). We further explore the correlations of *D*_app_ with light and wind speed by fitting for each day the 48 half hourly values of *D*_app_ versus PAR and wind speed. The correlation of *D*_app_ and wind speed was consistently positive for days with relatively low soil water content (<33%; Figure 4a, asterisks). Correlations between *D*_app_ and PAR were positive for almost all days (Figure 4b), indicating that *D*_app_ and thus the rate of CO_2_ transport increases with higher irradiation. Furthermore, the slopes of the 24 h fits of *D*_app_ versus PAR were positively correlated with wind speed (Figure 4c), indicating that the coupling of *D*_app_ and irradiation are amplified under windy conditions. To rule out any bias in either of our field measurement series, we subjected other data sets of combined solid‐state and chamber measurements, from the previous year and with different placement, to this analysis and found similar results (data not shown).

We tested how much of the overall variation in *D*_app_ could be attributed to variation in PAR, wind speed, and soil moisture, both for days above and below the threshold of 33% soil moisture content. The results of these multiple linear regressions are summarized in Table 1. For the entire data set, the combination of the three parameters explained 67% of the variation in *D*_app_. The combination of wind speed and PAR explained a larger part of the variation in *D*_app_ under moderately wet conditions (47%) than under very wet conditions (40%).

**Table 1:** Multiple Linear Regression of Wind Speed, Photosynthetically Active Radiation (PAR), and Soil Moisture Content (SWC) With Apparent Diffusivity (*D*_app_), Used to Calculate the Percentage of Variance in *D*_app_ Explained by Combinations of These Three Drivers^a^

	% Variance in *D*_app_ Explained		
Included Parameters	All data	SWC < 33%	SWC > 33%
Wind, PAR, and SWC	67	57	61
Wind and PAR	36	47	40

a Regressions were carried out on the whole data set, on the subset of data where SWC was below the threshold of 33% and on the subset of data where SWC was above this threshold.

**Figure 5:**
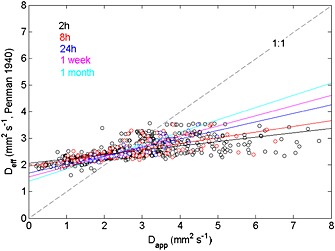
The accordance between *D_s_* (using the *Penman*'s [1940] model) and *D*_app_, with increasing integration times of solid state and chamber measurements: 2 h, 8 h, 24 h, 1 week, and 1 month.

Comparing half hourly values of soil CO_2_ concentrations with soil CO_2_ efflux measured once every half hour implies comparing different integration times. To avoid statistical bias related to a reduction in variability due to longer integration time, we tested how the soil CO_2_ efflux depended on the integration time of the flux‐gradient approach. To this end, we used the *Penman*'s [1940] equation, because it yielded the best result in predicting the effective diffusivity in the soil (see Figure 2). Increasing the integration time of the solid‐state and chamber measurements resulted in slightly improving accordance between *D*_app_ and *D_s_* (Figure 5), but even increasing the integration time to one month yielded poor agreement between *D*_app_ and *D_s_*.

## 4 Discussion

Soil CO_2_ concentrations and effluxes were studied at a mountain meadow in the Stubai Valley (Austrian Alps) during the period of peak biomass production in 2009. Diurnal variations in concentrations were small, while they were much larger for the chamber measurements, exceeding even the seasonal variability. Furthermore, when pooling all data pairs, both types of measurements seemed largely uncoupled, which is surprising given that the concentration difference of CO_2_ between soil and atmosphere is assumed to be the main driver of the soil CO_2_ efflux. Part of this uncoupling might be attributable to a time lag between photosynthesis and CO_2_ efflux, as reported by *Vargas et al*. [2011], but since such lag was not observed in the times series of soil CO_2_ concentration and efflux (Figure 1), we expect it to play a minor role here. The importance of a storage term was evaluated by looking at the instantaneous changes in soil CO_2_ concentrations, but as these turned out to be very small, a storage flux is unlikely to explain the uncoupling of concentration and flux measurements here. *Flechard et al*. [2007] pointed out that storage occurs only when diffusive transport prevails.

To evaluate the importance of nondiffusive transport mechanisms in determining the soil CO_2_ efflux at this site, we compared solid‐state CO_2_ fluxes, calculated with the flux‐gradient approach, to the chamber CO_2_ fluxes. The use of Fick's law in the flux‐gradient approach requires estimates of soil diffusivity that are in turn determined by the tortuosity factor. Different models exist for calculation of this tortuosity factor, all of them strongly depending on the air‐filled pore space and thus on the soil water content [*Werner et al*., 2004]. Among the models tested here, the *Penman* [1940] and *Marshall*'s [1959] models performed better with respect to the overall magnitude of the flux, the Penman's model also being the least sensitive to uncertainties in porosity estimates. The Penman's model yielded the highest soil CO_2_ effluxes, which in contrast to what has been frequently reported [*Pingintha et al*., 2010; *Sallam et al*., 1984; *Werner et al*., 2004], did not exceed chamber fluxes but predicted fluxes within the same range. The other three models resulted in fluxes (much) lower than those from chamber measurements. This was especially the case for the *Moldrup et al*. [1999] and *Millington and Quirk*'s [1961] model, although these have often been reported as yielding the best results [*Pingintha et al*., 2010; *Sallam et al*., 1984; *Werner et al*., 2004]. In our study, the most important outcome was that none of these diffusivity models was able to predict the short‐term (i.e., diel and day to day) variation of the soil CO_2_ efflux in an acceptable way (Figure 2).

Further analyses pointed out that this disagreement is related to the fact that the tortuosity models, based on diffusive transport, depend almost completely on the soil water content. The role of the moisture content on diffusive transport is twofold. First, by decreasing the air‐filled pore space, as diffusion of CO_2_ through water is much slower than diffusion through air. Second, there can be a hysteric effect of the water content on the effective diffusivity, *D_s_* being higher at a similar water content during wetting than during drying [*Goffin et al*., 2014; *Rouf et al*., 2012]. It is important to note here that the short‐term response of soil respiration to changes in soil moisture is also not monotonic; CO_2_ production increases from low to intermediate soil moisture, reaches a plateau at optimum moisture, and decreases again at high soil moisture [*Vicca et al*., 2014].

At our site, however, the rate of CO_2_ transport from soil to atmosphere depended not only on soil moisture but also on irradiation (PAR) and wind speed. The correlation coefficients of the fits of apparent diffusivity, *D*_app_, versus wind speed were consistently positive on days that were not very wet, i.e., when soil water content was below 33% (Figure 4a). *D*_app_ was also positively related to PAR (Figure 4b), indicating a positive light effect on CO_2_ transport from soil to atmosphere. Last, the slopes of the linear regressions showed that the coupling of *D*_app_ and PAR became stronger as wind speed increased (Figure 4c). Note that higher values of PAR and wind speed were recorded in periods when soil moisture content was below 33%, therefore leading also to higher values of *D*_app_ in these periods. The response of *D*_app_ to these drivers, on the other hand, did not differ for periods with soil moisture content above and below 33% (intercepts of the correlations differed but not the slopes). The effect of wind speed on *D*_app_ was similar during day and nighttime.

The large diel variations we observed in *D*_app_ and its tight coupling to PAR should not be mistaken for a soil respiration‐driven effect on diffusive CO_2_ transport. Soil respiration is known to be strongly influenced by temperature and thus exhibits a pronounced diel pattern. This diel pattern can be further amplified by the linkage between belowground carbon allocation and photosynthetic activity. The increase in photoassimilates allocated below ground was often shown to stimulate autotrophic and heterotrophic respiration [*Bahn et al*., 2009; *Hogberg et al*., 2001; *Janssens et al*., 2001; *Kuzyakov*, 2006; *Kuzyakov and Gavrichkova*, 2010].

The diurnal pattern in soil respiration was clearly seen in the chamber‐based soil CO_2_ efflux measurements, but surprisingly much less in the soil CO_2_ concentration measurements. This suggests that an increased transport rate, exceeding diffusion, during the day must have prevented soil CO_2_ concentrations to rise, while soil respiration did increase. Diffusion primarily depends on soil moisture and on the concentration gradient, both of which varied only little during the day. Enhanced transport of CO_2_ through the soil via other processes should thus explain the discrepancy between the large diel amplitude of soil CO_2_ efflux and the relatively steady soil CO_2_ concentrations.

Several mechanisms causing the bulk flow of CO_2_‐enriched soil air have been described in literature (see *Kuang et al*. [2013] for an extensive review) and can occur in all soils when pores are connected and not blocked by water [*Cuezva et al*., 2011]. An increasing number of authors have demonstrated a positive correlation between pressure pumping and soil CO_2_ efflux [*Arneth et al*., 1998; *Baldocchi and Meyers*, 1991; *Lewicki et al*., 2010; *Subke et al*., 2003]. *Takle et al*. [2004] found that these pressure fluctuations penetrated into a dry soil up to 50 cm with little attenuation, and *Bowling and Massman* [2011] found a temporarily transport enhancement through a forest snowpack up to 40% higher than diffusion. Pressure pumping is controlled by the degree of permeability of the medium and the direction and magnitude of the pressure gradient [*Massman et al*., 1995; *Takle et al*., 2003]. Pressure gradients can be caused by barometric waves, passage of synoptic weather systems, short‐period atmospheric turbulence, and wind blowing across irregular topography [*Elberling et al*., 1998; *Massman et al*., 1997; *Takle et al*., 2004].

Bulk air transport (in this context called ventilation) was described in permeable, dry, and fractured media, such as karst systems, and was found to be coupled to pressure gradients as well as wind [*Rey et al*., 2012; *Sanchez‐Canete et al*., 2013; *Serrano‐Ortiz et al*., 2010].

The bulk exchange of gases is enhanced when drying of the soil increases the air‐filled porosity and by high wind speeds [*Hirsch et al*., 2004; *Kimball and Lemon*, 1971; *Maier and Schack‐Kirchner*, 2014; *Sanchez‐Canete et al*., 2013; *Subke et al*., 2003]. The positive correlation that we found between *D*_app_ and wind under relatively dry conditions could therefore be attributable to pressure changes caused by wind shear at the soil surface.

Advective bulk air transport can also be triggered by the local heating of the soil surface [*Ganot et al*., 2014]. This heating would not be recorded by the soil temperature sensors, which were installed at 5 and 10 cm depths, but inevitably coincides with measurements of irradiation. A second light‐coupled process that may amplify bulk soil air transport is the breaking of the soil boundary layer following thermal expansion and uplifting of the air at the soil‐atmosphere interface.

Several things could improve the ability to accurately estimate CO_2_ efflux from vertical concentration gradients. First, *D_s_*, and thus porosity and tortuosity, should be independently estimated using intact soil cores [*DeSutter et al*., 2008; *Jassal et al*., 2005] or in situ field methods based on inert tracers such as radon [*Risk et al*., 2008], although even these methods cannot fully capture the spatial and temporal dynamics of the soil gas diffusivity [*Maier and Schack‐Kirchner*, 2014]. Next, part of the discrepancy that we observed between solid‐state and chamber CO_2_ efflux might be reconciled when both types of measurements are carried out with the exact same measuring frequency and interval, as the variability of solid‐state concentrations with longer integration time will typically be lower otherwise. This was also brought up by *Riveros‐Iregui et al*. [2008], stating that solid‐state sensors might not capture rapid changes in soil properties and respiration, e.g., due to rainfall events. Several authors state that the efflux rates calculated from concentration gradients might be unsuited for deriving short‐term fluxes, but that they could be useful for flux estimates over longer time periods [e.g., *Vargas et al*., 2010] when the importance of nondiffusive transport decreases. However, even after integration over 1 month, we found that apparent diffusivity was higher than effective diffusivity (Figure 5), thus still resulting in an underestimation of soil respiration rates by the flux‐gradient approach. Substantial uncertainty may furthermore arise from the assumption of a linear CO_2_ concentration gradient in the soil [*Maier and Schack‐Kirchner*, 2014], although *Monson et al*. [2006] found it a minor source of error compared to the estimations of *D_s_*. The exact measurement height of the 0 cm concentration holds a negligible error, given that the magnitude and diel variability of soil CO_2_ above the surface are small due to the atmospheric buffer and affects the gradient only very little [*Riveros‐Iregui et al*., 2008, and own analysis].

Several previous studies did find good agreements between solid‐state CO_2_ efflux and chamber CO_2_ efflux [*Jassal et al*., 2005; *Liang et al*., 2004; *Pumpanen et al*., 2008; *Tang et al*., 2005]. These studies were conducted in dense forests, where in contrast to grasslands, both light and wind have difficulty penetrating into layers close to the soil surface. This is probably why their effect on soil gas transport was insignificant. Under such diffusion‐dominated conditions, the flux‐gradient approach can be successfully applied to predict the soil CO_2_ efflux.

## 5 Conclusions and Recommendations

Over the last decade, research in this field has focused on the measurement of fluxes at the soil surface using a variety of chambers and micrometeorological methods. Recently, the flux‐gradient approach proved to be a very cost‐efficient way to calculate the soil CO_2_ efflux that minimizes soil surface perturbations and provides insights into subsurface CO_2_ dynamics. This method uses estimates for effective diffusivity, derived from different models primarily based on the air‐filled pore space. Testing four commonly used models, we found substantial deviation between observed and modeled diffusivities, leading to poor predictions of the soil CO_2_ efflux when using the flux‐gradient approach at shorter time scales.

Gaseous diffusion is often considered as the only mechanism for CO_2_ to move from the soil to the atmosphere, while in reality, several gas transport mechanisms can be distinguished in unsaturated porous media like soils, e.g., advective mass transport and pressure pumping. We found strong evidence for such nondiffusive CO_2_ transport at our site, given that the rate of transport was coupled to irradiation, with an even stronger coupling under increasing wind speed. Wind speed also had a direct positive effect on the efflux rate when the soil moisture content is comparatively low (<33%), resulting in higher air‐filled porosity.

Considering the importance of alternative transport processes is a prerequisite when using solid‐state CO_2_ concentration measurements to estimate soil CO_2_ efflux at any given site. Deviation between apparent (data‐based) and effective (model‐based) diffusivities may be a first indication of nondiffusive gas transport. Apparent diffusivity can be evaluated by calculation from combined chamber and solid‐state sensor measurements (as demonstrated here) or by direct in situ soil gas diffusivity measurements (e.g., with natural or injected ^222^Radon as done by *Risk et al*. [2008]).

Given that nondiffusive transport is especially important at very short time scales and its influence decreases with increasing time scale, we recommend that future studies further explore the importance of the time scale considered (high‐frequency versus low‐frequency data) and the effects of uncertainties in porosity on this. Combined high‐frequency measurements of soil CO_2_ concentrations and its potential physical drivers, including air pressure and friction velocity, under a wider range of soil moisture conditions, are needed to obtain a more detailed picture of the mechanisms and time scales relevant for nondiffusive transport of CO_2_ on soils.
